# Mixed Red-Complex Bacterial Infection in Periodontitis

**DOI:** 10.1155/2013/587279

**Published:** 2013-03-06

**Authors:** N. Suzuki, M. Yoneda, T. Hirofuji

**Affiliations:** ^1^Section of General Dentistry, Department of General Dentistry, Fukuoka Dental College, 2-15-1 Tamura, Sawara-ku, Fukuoka 814-0193, Japan; ^2^Center for Oral Diseases, 3-2-1 Hakataekimae, Hakata-ku, Fukuoka 812-0011, Japan

## Abstract

The red complex, which includes *Porphyromonas gingivalis*, *Treponema denticola*, and *Tannerella forsythia* (formerly *Bacteroides forsythus*), are recognized as the most important pathogens in adult periodontal disease. These bacteria are usually found together in periodontal pockets, suggesting that they may cause destruction of the periodontal tissue in a cooperative manner. This article discusses the interspecies pathogenic interactions within the red complex.

## 1. Introduction

Periodontal diseases are polymicrobial immune-inflammatory infectious diseases that can lead to the destruction of periodontal ligaments and adjacent supportive alveolar bone. The subgingival plaque contains more than 700 bacterial species, and some of these microorganisms have been shown to be responsible for initiation/progression of periodontal diseases [1, 2]. The red complex, which includes *Porphyromonas gingivalis*, *Treponema denticola*, and *Tannerella forsythia *(formerly *Bacteroides forsythus*), encompasses the most important pathogens in adult periodontal disease [3]. Additionally, *Fusobacterium nucleatum*, *Prevotella *species, *Eikenella corrodens*, *Peptostreptococcus micros*, and *Campylobacter rectus *are increased in deep periodontal pockets and are implicated as possible periodontopathogens [1–4]. These bacteria are not usually found alone, but in combination in the periodontal pockets, suggesting that some bacteria may cause destruction of the periodontal tissue in a cooperative manner [5]. Studies using animal models have reported the synergistic pathogenicity of mixed infections with *P. gingivalis*-*T. denticola*, *P. gingivalis*-*F. nucleatum*, *P. gingivalis*-*T. forsythia*, *P. gingivalis*-*Aggregatibacter actinomycetemcomitans*, *F. nucleatum*-*T. forsythia*, and *P. gingivalis*-*T. denticola*-*T. forsythia* [6–11]. Furthermore, coaggregation, nutrient effects, and modulation of virulence factors by periodontopathogens or by interspecies interactions between periodontopathogenic and nonpathogenic organisms have been reported to contribute to oral microbial pathogenesis [12]. This paper focuses on interspecies pathogenic interactions within the red complex, in particular the combinations of *P. gingivalis-T. forsythia*, *P. gingivalis-T. denticola*, and *P. gingivalis*-*T. forsythia*-*T. denticola*. Potential therapies using normal inhabitants of the oral microbiota that have an antagonistic relationship with the red complex are discussed. 

## 2. *P. gingivalis* and *T. forsythia*

*P. gingivalis *possesses many virulence factors, such as fimbriae, lipopolysaccharides, and proteases [13–15]. The arg-gingipain (Rgp) and lys-gingipain (Kgp) cysteine proteinases are important for the virulence of *P. gingivalis *as they elicit dysfunction of inflammatory and immune responses and can degrade various connective tissue proteins [16, 17]. Rgp is encoded by two separate genes (*rgpA *and *rgpB*), whereas Kgp is encoded by a single gene (*kgp*) [18]. In a murine abscess model, *rgpA *and *rgpB *double and *kgp *single-mutants induced smaller abscesses than did the wild type, and the *rgpA-rgpB-kgp *triple (gingipain-null) mutant showed negligible lesion formation [19]. These findings indicate that gingipains play an important role in abscess formation in mice. 

Compared to the abscess formation induced by monoinfection with each bacterium in the abovedescribed murine model, mixed infection with wild-type *P. gingivalis *ATCC 33277 and *T. forsythia *ATCC 43037 showed a synergistic effect on abscess formation [19]. On the other hand, mixed infection with *P. gingivalis *mutants devoid of gingipain (*rgpA rgpB*, *kgp*, and *rgpA rgpB kgp*) with *T. forsythia *showed only just an additive effect on abscess formation. These findings suggest that the gingipains of *P. gingivalis* play an important role in the pathological synergism between *P. gingivalis *and *T. forsythia*. A combination of *T. forsythia *strains isolated from periodontitis patients and *P. gingivalis* also showed synergistic pathogenesis in a rabbit abscess model [20]. The researchers found 100% abscess formation in rabbits with mixed infection using *P. gingivalis *and *T. forsythia* but 0% in rabbits with monoinfection with each bacterium. Recently, Verma et al. [21] injected *P. gingivalis *FDC 381 and *T. forsythia *ATCC 43037 into the oral cavity of rats and evaluated the synergistic effect of mixed infection on periodontal disease. Mixed colonization by *P. gingivalis *and *T. forsythia *in the rat oral cavity was confirmed by polymerase chain reaction (PCR). The induction of moderate periodontal inflammation and pronounced apical migration of junctional epithelium, generation of a specific immunoglobulin G (IgG) antibody response, and stimulation of both Th1- and Th2-like immune responses as reflected by the serum IgG subclass profiles were evident. Mixed infection with *P. gingivalis *and *T. forsythia *resulted in a significant increase in interproximal alveolar bone resorption compared to that in rats infected with a single bacterial strain and controls, but there was no synergy between the bacteria [21]. The researchers indicated that the virulence of *P. gingivalis *and *T. forsythia *mixed infection resulted from the immune-inflammatory responses and the lack of humoral immune protection during periodontitis in rats. 

Humoral immune responses were induced by *P. gingivalis *in the murine abscess model [22]. Mice that were first infected with a wild-type strain and subsequently reinfected with the same wild-type strain showed significantly smaller lesions than control mice that were mock-infected with medium only and then re-infected with the wild-type strain. Yoneda et al. [23] assessed the serum IgG antibody response in a murine abscess model after mixed infection with various levels of *P. gingivalis *ATCC 33277 or *T. forsythia *ATCC 43037 whole bacteria cell antigens. After mixed infection, IgG antibody responses to *P. gingivalis *increased in proportion to the level of *T. forsythia *injected. In contrast, IgG antibody responses to *T. forsythia *did not correlate with the level of *P. gingivalis *injected. Reasons for this difference may be that *P. gingivalis *and *T. forsythia *induced different antibody responses after mixed infection in mice; alternatively, these bacteria may exhibit different interactions in terms of growth at the injected sites. 

*T. forsythia*, which is a fastidious anaerobic Gram-negative rod, is frequently isolated together with *P. gingivalis*, especially from the active state of periodontitis [24–27]. It is well known that the growth of *T. forsythia *is accelerated on blood agar when cocultivated with *P. gingivalis *or *F. nucleatum*, suggesting that a form of symbiosis occurs with respect to nutrition [28]. Furthermore, the growth-promoting factors appear to be proteinaceous in nature. Sonicated cell extracts of *T. forsythia *ATCC 43037 stimulate growth of *P. gingivalis *ATCC 33277 in nutrition-decreased medium in a dose-dependent manner [29], whereas the cell extract of *T. forsythia *had no stimulatory effect on the growth of the *rgpA rgpB kgp *triple (gingipain-null) mutant of *P. gingivalis*. These results suggest that gingipains of *P. gingivalis *play an important role in the digestion or uptake of the growth-promoting factor derived from *T. forsythia*. The growth-promoting interaction between *P. gingivalis *and *T. forsythia* may be partly related to synergistic virulence in a murine abscess model. 

*T. forsythia *is a member of the polymicrobial flora that invades buccal epithelial cells taken directly from the mouth [30]. Epithelial cell invasion by periodontopathogens is considered to be an important virulence mechanism for evasion of the host defense responses and for forming reservoirs important in recurrent infections. *T. forsythia *possesses some putative virulence factors, such as a trypsin-like protease, a sialidase, hemagglutinin, components of the bacterial S-layer, and a cell surface-associated and secreted protein (BspA) [31]. BspA has been recognized as a virulence factor important for alveolar bone loss in mice [31]. Inagaki et al. [32] investigated the epithelial cell adherence and invasion abilities of *T. forsythia *and reported that these are dependent on BspA. Additionally, they found that *P. gingivalis* FDC 381 or its outer membrane vesicles enhance the attachment and invasion of *T. forsythia *ATCC 43037 to epithelial cells. 

## 3. *P. gingivalis* and *T. denticola*

*T. denticola*, a small oral spirochete, is frequently found with* P. gingivalis *in progressing periodontitis lesions [1, 33–35]. *T. denticola *is located within the surface layers of the subgingival plaque, whereas *P. gingivalis *is observed predominantly beneath the spirochete layer [33]; a symbiotic nutrient utilization relationship between these two periodontopathogens has been shown *in vitro* [36]. The growth-stimulating factors produced by *P. gingivalis *ATCC 33277 and *T. denticola *ATCC 35405 have been identified as isobutyric acid and succinic acid, respectively [36]. 

Biofilm formation is also an important step in the etiology of periodontal diseases. *P. gingivalis*, but not *T. denticola *strains, formed biofilms *in vitro *[37]. Coculture of *P. gingivalis *FDC 381 and *T. denticola *ATCC 35405 induced synergistic biofilm formation and coaggregation. Confocal microscopy demonstrated that *P. gingivalis* attaches to the substratum first as the primary colonizer followed by coaggregation with *T. denticola *to form a mixed biofilm [38]. The *T. denticola *flagellar mutant and cytoplasmic filament mutant exhibit significantly reduced biofilm formation with *P. gingivalis*. Similarly, the *P. gingivalis *gingipain mutant and major fimbriae mutant exhibited significantly reduced biofilm formation with *T. denticola *[38]. Using two-dimensional electrophoresis followed by a ligand overlay assay with *P. gingivalis *fimbriae, Hashimoto et al. [39] determined that dentilisin, a chymotrypsin-like proteinase of *T. denticola*,  was the *P. gingivalis *fimbriae-binding protein. These results support the hypothesis that these two organisms assist each other's survival in subgingival sulcus and explain why they are frequently isolated together from subgingival plaque.

Synergistic virulence of mixed *P. gingivalis *and *T. denticola *infection has been assessed in several animal lesion models. Using a murine abscess model, one group reported that high doses of *P. gingivalis *W50 (1-2 × 10^10^ cells per dose) together with *T. denticola *ATCC 35404 (1 × 10^9^ or 1 × 10^10^ cells per dose) had no effect on the formation and size of the spreading lesion caused by *P. gingivalis* [40]. However, at low *P. gingivalis* doses (1-2 × 10^9^ cells), addition of *T. denticola *(1 × 10^10^ cells) significantly enhanced the virulence of *P. gingivalis *compared with monoinfection [40]. Investigation of the synergistic virulence of *P. gingivalis *and *T. denticola *using a murine experimental model of periodontitis found that a 1 : 1 ratio of *P. gingivalis *W50 and *T. denticola *ATCC 35405 coinoculum at 5 × 10^8^ or 1 × 10^9^ total bacterial cells induced the same level of bone loss as four doses of 1 × 10^10^ *P. gingivalis *[41]. Coinoculation induced strong *P. gingivalis*-specific T cell proliferative and interferon-*γ* (IFN-*γ*) cytokine responses and induced a strong *T. denticola*-specific IFN-*γ* cytokine response. Another study using a rat model of periodontal disease reported that a mixed *P. gingivalis *FDC 381 (5 × 10^9^ cells) and *T. denticola *ATCC 35404 (5 × 10^9^ cells) infection produced significantly more interproximal and horizontal alveolar bone loss compared to monoinfections (1 × 10^10^ cells); however, there was no synergy between *P. gingivalis *and *T. denticola *[42]. Furthermore, colonization of these bacteria was observed in the rat oral cavity during 7 weeks of periodontal disease, resulting in the generation of a specific serum IgG antibody response that reflected oral infection and the induction of an inflammatory response consistent with the established characteristics of periodontitis. These results suggest that *P. gingivalis *and *T. denticola *act synergistically (with no synergy between the bacteria) to stimulate the host immune response and to induce alveolar bone loss in a rat model of periodontitis [42]. 

## 4. *P. gingivalis*, *T. forsythia*, and *T. denticola*

Epithelial cells and macrophages play a major role in the host response to periodontopathogens, and secretion of inflammatory mediators and matrix metalloproteinases (MMPs) by these host cells contribute to periodontal tissue destruction. Bodet et al. [43] investigated the inflammatory response of an *in vitro* macrophage/epithelial cell coculture model following mono- or mixed infections with whole bacterial cells of the red-complex and their lipopolysaccharide (LPS). Mono- or mixed infections of the coculture model induced the secretion of interleukin-1 beta (IL-1*β*), IL-6, IL-8, prostaglandin E_2_ (PGE_2_), and MMP-9. All LPS mono- or mixed infections induced an increase in chemokine, MMP-9, and PGE_2_ production. Compared to mono-infections with individual bacterial species, no synergistic effects on cytokine, PGE_2_, or MMP-9 production by the bacterial mixtures tested were observed. *P. gingivalis *and *T. forsythia *induced the secretion of RANTES (regulated and normal T cell expressed and secreted), whereas *T. denticola *alone or in combination with *P. gingivalis *resulted in a significant decrease in RANTES levels. RANTES degradation by mono- or mixed infections with red-complex bacteria resulted in massive proteolytic degradation of RANTES by *T. denticola*. The ability of periodontopathogens to degrade cytokines and chemokines *in vivo *may play an important role in their pathogenicity by disrupting the host inflammatory response. 

Recently, polymicrobial infection with *P. gingivalis*, *T. denticola*, and *T. forsythia *in a rat model of periodontal disease was investigated [44]. A 1 × 10^10^ cell mixture (1 mL) containing an equal number of cells of each bacterium was injected into the rat oral cavity. PCR of the bacterial DNA in the oral sample revealed that polymicrobial infection enhanced colonization by *P. gingivalis*, *T. denticola*, and *T. forsythia* compared to their levels in monomicrobial infections. Oral infection of rats with a polymicrobial consortium comprising *P. gingivalis*, *T. denticola*, and *T. forsythia *induced significant increases in maxillary and mandibular alveolar bone resorption compared to those resulting from any of the monomicrobial infections (*P* < 0.001). The levels of serum IgG against all of the bacteria in the polymicrobial infection were lower than the respective levels induced by monomicrobial infections. This suggested that the host response to the polymicrobial infection was altered, resulting in enhanced evasion of protective immune responses by the bacterial consortium. 

## 5. Future Directions of Periodontitis Therapy

Most therapeutic modalities for treatment of periodontitis aim to remove pathogens and kill all bacteria in the periodontal pocket. Understanding the effect of interbacterial interactions on the pathogenesis of periodontitis may facilitate development of novel treatment modalities, such as the inhibition of adherence using antagonists, passive immunization, replacement therapy, regulation of levels of nonpathogenic bacteria to modulate virulence, probiotics, and interference with signaling mechanisms [12]. The disruption of the harmonic relationship between the host and commensal microorganisms is considered to be an important factor for the development of oral pathologies. The microbiota composition of healthy and periodontitis patients differs [45–47]. Here, we discuss the significance of interbacterial antagonism for maintenance and recovery of a healthy oral microbiota. Antagonistic bacteria present a substantial barrier to colonization by exogenous and opportunistic endogenous pathogens. Moreover, antagonistic bacteria have the potential for probiotic action, which may protect against periodontitis. 

Studies have identified organisms antagonistic to periodontopathogens. With regard to the red complex, *Staphylococcus aureus *and *Streptococcus mutans *isolates inhibited the growth of *T. denticola *and *P. gingivalis *[48]. *S. aureus* strains produced a bacteriocin-like inhibitory substance, whereas the inhibitory effect of *S. mutans *was related to the production of lactic acid. *S. sanguinis*, *S. cristatus*, *S. salivarius*, *S. mitis*, *Actinomyces naeslundii*, and *Haemophilus parainfluenzae *inhibited the adhesion of standard *P. gingivalis *strains *in vitro* [49]. *S. cristatus *arginine deiminase repressed FimA, a major subunit protein of the long fimbriae, and inhibited biofilm formation by *P. gingivalis *[50].

Other studies have identified organisms with probiotic potential against periodontopathogens. An analysis of the ability of clinical isolates from healthy and periodontitis patients to inhibit the growth of periodontopathogens showed that the number of isolates from healthy volunteers that inhibited either *P. gingivalis *or *P. intermedia* was significantly higher than that from diseased patients [51]. These isolated growth-inhibiting strains included some viridans group *Streptococcus*, *Actinomyces*, and *Bifidobacterium* strains. Compared to these isolates, commercial dietary probiotics showed stronger inhibition of the periodontopathogens. In a study that compared oral lactobacilli from patients with chronic periodontitis and periodontally healthy subjects, the most prevalent species in healthy subjects were *Lactobacillus gasseri *and *L. fermentum*, whereas the most prevalent species in subjects with periodontitis was *L. plantarum *[52]. Furthermore, the greatest antimicrobial activities were associated with *L. paracasei*, *L. plantarum*, *L. rhamnosus*, and *L. salivarius*. The international guidelines for the evaluation of probiotics confirm that these four organisms exhibit both high antimicrobial activity and high tolerance to environmental stress [53]. 

Some oral bacteria possess the potential for antagonism towards periodontopathogens, which highlights the therapeutic potential of stimulation of oral health using beneficial effector strains. Most evidence indicates that probiotics in the gut do not populate the gastrointestinal microbiota permanently, and they disappear from feces soon after cessation of probiotic ingestion [54]. Similarly, altering the composition of established oral plaque microbial communities is difficult [55]. Probiotic bacteria used in the human oral cavity include *Bifidobacterium *and *Lactobacillus *species, and most of them were not derived from the oral cavity [56]. *L. reuteri *and *L. salivarius *colonized the oral cavity of patients; however, the study was of only 2 weeks duration [57–59]. Another study reported that oral administration of *L. salivarius* decreased the proportion of *L. salivarius *in saliva during the 4- and 8-week intervention periods, although the sampling and analysis methods differed [60]. Organisms antagonistic to periodontopathogens that are derived from typical representatives of the oral microbiota and have probiotic potential may overcome the weaknesses associated with exogenous probiotic bacteria. 
